# HS-SPME-GC–MS Volatile Profile Characterization of Peach (*Prunus persica* L. Batsch) Varieties Grown in the Eastern Balkan Peninsula

**DOI:** 10.3390/plants11020166

**Published:** 2022-01-08

**Authors:** Dasha Mihaylova, Aneta Popova, Radka Vrancheva, Ivayla Dincheva

**Affiliations:** 1 Department of Biotechnology, Technological Faculty, University of Food Technologies, 4002 Plovdiv, Bulgaria; 2 Department of Catering and Nutrition, Economics Faculty, University of Food Technologies, 4002 Plovdiv, Bulgaria; 3 Department of Analytical Chemistry and Physical Chemistry, Technological Faculty, University of Food Technologies, 4002 Plovdiv, Bulgaria; radka_vrancheva@yahoo.com; 4 AgroBioInstitute, Agricultural Academy, 1164 Sofia, Bulgaria; ivadincheva@yahoo.com

**Keywords:** *Prunus persica* L., gas chromatography–mass spectrometry (GC–MS), volatile compounds, principal component analysis (PCA), hierarchical cluster analysis (HCA), headspace-solid phase micro extraction (HS-SPME)

## Abstract

The volatile compounds of eight peach varieties (*Prunus persica* L.)—“Filina”, “Gergana”, “Ufo-4”, “July lady”, “Laskava”, “Flat Queen”, “Evmolpiya”, and “Morsiani 90”—growing in Bulgaria were analyzed for the first time. Gas chromatography–mass spectrometry (GC–MS) analysis and the HS-SPME technique revealed the presence of 65 volatile compounds; the main identified components were aldehydes, esters, and fatty acids. According to the provided principal component analysis (PCA) and hierarchical cluster analysis (HCA), the relative quantities of the identified volatile compounds depended on the studied peach variety. The results obtained could be successfully applied for the metabolic chemotaxonomy of peaches.

## 1. Introduction

The diversity of volatile compounds is responsible for the unique flavors each food matrix expresses. Aroma is a particularly important and valued feature that illustrates the complex mixture of volatile compounds in foods. The human nose can sense a broad selection of volatile compounds. Although many studies focus on the volatile profiles of various fruit and vegetables, there is a particular need to enhance the available information.

Peaches and nectarines are aroma-dense fruits with a specific, pleasant, and recognizable aroma [1]. Nectarines (*Prunus persica* var. nectarina) may have developed from peach seeds, but their origin is still unknown. The peach (*Prunus persica* L. Batsch), also known as Persian apple, is native to China and Iran. Subsequently, it has spread worldwide. Peaches have a large number of commercial varieties with different shapes, sizes, flesh colors (red, white, or yellow), skin types, seeds, among other variable aspects in relation to this popular fruit, representing a diverse international germplasm [2,3]. The largest producer is China, followed by Italy, Spain, and the United States [2].

The peach is a widely appreciated fruit for consumption, but has not yet been fully studied. The chemical composition of peaches depends on several factors, such as genotype, geographical and climatic conditions, seasonal and meteorological conditions, agronomic practices, stage of maturity, storage conditions, and processing methods [4]. In addition, it has been shown, over the years, that the phytochemicals are not evenly distributed in the fruit tissue; most are concentrated in the rind, particularly in the epidermal and subepidermal layers [5,6,7].

Volatile compounds, together with sugars and acids, are the main chemical compounds that determine the characteristic aroma and flavor of foods. The peach species holds remarkable characteristics. More than a hundred volatiles have been identified in different peach varieties, with C_6_ compounds, esters, benzaldehyde, linalool, C_13_ norisoprenoids, and lactones being the most abundant [8,9]. In general, polyunsaturated fatty acids (PUFAs), such as linoleic acid (18:2) and linolenic acid (18:3), are the main precursors for aroma-related volatiles of peach fruit generated via the lipoxygenase (LOX) pathway or β-oxidation [10]. β-Oxidation leads to the production of the primary aroma in fruits, whereas the LOX system may account for the widest assortment of lipid-derived precursors of aroma compounds in disrupted plant tissues [11]. In addition to their contribution to fruit quality, peach volatiles are also important for the food and fragrance industry, where they are used as flavoring agents. A notable example of a sought-after industrial product with a peach-like aroma is γ-decalactone [12].

Numerous studies demonstrated the large variability in the volatile compound composition of peaches depending on the cultivar, ripening stage, and geographical origin [13,14,15]. Nectarines generate fewer volatiles than peaches, but have more fruity and floral aroma notes due to greater ester, linalool, and terpinolene production [16]. Authors report changes in volatile aroma-related substances during peach fruit development and ripening after harvest [17,18,19].

Although the volatile profiles of peaches have been generally widely studied, no information exists on Bulgarian peach varieties. Thus, the goal of this study was to investigate the difference in volatile profiles between eight peach and nectarine varieties in order to provide a tool to evaluate and compare the data on volatiles.

## 2. Results and Discussion

Peach fruit produces a number of volatile organic compounds (VOCs) [20,21]. The investigation of different peach varieties showed that C_6_ compounds, alcohols, aldehydes, and lactones are the most powerful aroma-active compounds [13,14]. Nectarines usually contain C_9_ aldehydes, γ-decalactone, and terpenes; peaches contain a majority of C_6–10_ lactones; and flat peaches are dominant in benzaldehyde, γ-decalactone, and δ-dodecalactone [22].

It is no coincidence that volatile compounds are of scientific interest. A quantitative comparison is not always feasible because of the variations in the extraction procedures and quantification procedures used. Different conclusions can be made based on the methods used for collection, the compound concentrations, and the nature of the volatiles produced by fruit. A recent study confirmed significant differences in the number of identified compounds and their quantities exist [23].

The typical taste of most fruits is not present during early formation, but develops after the ripening process [21]. During this period, metabolism changes to catabolism and volatile compounds are formed from the main plant constituents by various biochemical pathways [24]. The climacteric respiration of the fruit aids in the flavor formation during the post-climacteric maturation phase [25]. The richness of the aromatic compounds is of primary importance for consumer acceptance.

Many researchers have confirmed that the same cultivars act differently when subjected to another environment. The varieties “Morsiani 90”, “Ufo- 4”, “July Lady”, and “Flat Queen” are varieties that have been introduced to the Bulgarian geographical region, which means that the orchard management and the ecological factors can result in a different VOCs profile. “Evmolpiya”, “Laskava”, “Filina”, and “Gergana”, on the other hand, are local varieties, and their volatile profile is yet to be reported. “Filina” is a result of the breeding selection of “Maycrest” × “July Lady”. “Gergana” is created by combining Goldengrand and Aureliogrand varieties. “Laskava” is created by cross-species hybridization, with the participation of the species *Prunus persica* L. Batsch and *Prunus ferganensis* (Kost. and Rjab.) from the parent combination “Hale” × (“Elberta” × “Fergana Yellow”). “Evmolpiya” is a variety obtained by the interspecific hybridization with the participation of *P. persica* var. nucipersica, *P. persca* and *P. davidiana*, from the parent combination “Fantasia” × (“Halle” × *Prunus davidiana*).

### 2.1. Gas Chromatography–Mass Spectrometry (GC–MS) Profiling of Volatile Compounds of Analyzed Peach Samples

The volatile profiles of eight peach varieties (four local and four introduced) grown in Bulgaria were analyzed by GC–MS. Table 1 is a visual presentation of the results; sixty-five volatile compounds, belonging to seven chemical classes (aldehydes, ketones, alcohols, fatty acids, esters, hydrocarbons, and terpenes), were identified.

Aldehydes comprise 21% of the identified compounds, with the dominance of hexanal, (E)-2-hexenal, and nonanal in all peach varieties (Table 1). Some aldehyde compounds are formed in the event of frost damage: octanal, heptanal, and pentanal [26]. The different amounts in the studied samples prove that chilling injuries are variety-dependent, and do not follow the ripening period of the peach.

Aldehydes are flavor-contributing for premature fruit. They bring out a specific fresh-green odor to the fruit. C_6_ aldehyde compounds are desired, especially in not fully ripe pears, plums, and apples. Such compounds decrease in quantity during the process of full ripening of the fruit [27]. Following the abovementioned, it can be concluded that in the absence of other unfavorable conditions, the “Laskava” variety, which contains the most aldehydes, can be stored for the longest period, while “Ufo-4” and “Evmolpiya” should reach the market within the shortest time. The melon-like flavor of the “Laskava”, “Ufo-4”, and “July Lady” varieties could be related to (E)-2-nonenal detection [28]. Hexanal is reported in literature [29] as a major compound in the volatile analysis of nectarines, which is further supported in the currently established results for the “Gergana” and “Morsiani 90” varieties. It is associated with a sweet, fruity taste [30]. Heptanal, 2-hexenal, and octanal, typical for peach varieties, were found to contribute to the fresh odor [31].

Lactones possess high aromatic values in peaches due to their low odor threshold. Lactones, as intramolecular esters of 4- and 5-hydroxy acids, shape the basic peach aroma [22], and have high aroma effects in stone fruits, in general. Among the seven identified ketones, γ-octalactone and γ-dodecalactone, that give peach-like aroma, were in the highest relative concentrations. These lactones act in association with aldehydes, alcohols and terpenoids, which are responsible for the spicy, floral and fruity features in the peach [32]. γ-Decalactone and γ-octalactone are characteristic volatile compounds for peaches. The compound γ-octalactone, which confers a sweet herbaceous, coconut-like odor and taste, was predominant in the “July Lady” and “Laskava” varieties [33]. γ-Decalactone was most abundant in the two nectarine varieties object of analysis, which supports the literature stating that this is the most common compound identified in the pulp of nectarines [13]. It has to be noted that lactone identification is highly dependent on the extraction conditions, which can be identified as a limitation in every study on the subject. The absence or presence of certain lactones can be due to the assessment methodology being used [34,35]. For example, other authors have managed to identify more than ten C_5_-C_10_ γ-lactones [8,36]. It has been suggested that lactones in peaches are a result of the β-oxidation pathway of fatty acids [21].

The biosynthesis of fatty acids has been reported to being highly influential on the volatile profile. Eight fatty acids were identified, with hexanoic acid (1.22–6.84% of TIC) being the principle one (Table 1). Nonanoic and dodecanoic acid were the second most abundant of the investigated peach fruits. Fatty acids are important as they serve as carriers for some lipophilic vitamins and bioactive compounds present in fruits, and the presence of essential fatty acids is believed to play an important role in the prevention of cardiovascular diseases [37,38]. Acids most likely contribute little to the aroma profile though, because they normally have high odor detection thresholds [39].

Alcohols represent approximately 8% of the total identified compounds, with pentanol and nonanol predominating. Pentanol, which is responsible for the bouquet and astringent aroma description [40], was the main alcohol in all the studied samples (in the range from 0.95% to 1.88% of TIC). Benzyl alcohol, found in the highest concentrations in “Ufo-4”, is described as having a floral aroma [41]. Alcohol dehydrogenase in the fruit mesocarp accumulates throughout ripening [20], and alcohols are usually left undetectable by the consumers. Relatively low alcohol quantities suggest that the fruit is not overripe [42]. Fruit juice pH effectively converts alcohols and aldehydes into flavoring agents [42].

Esters are the main VOCs produced by horticultural crops. Esters, especially straight chain esters, are generally metabolized from fatty acids [43]. The higher the amount of esters, the more pronounced the aroma and the taste of the fruit [44]. Esters, accounting for 25% of the identified compounds, represented the largest group. The composition of esters differed both qualitatively and quantitatively among the peach samples. It has to be noted that the ester distribution is reported to be different within the part of the fruit [36]. The total ester content varied between 25.51% and 34.61% of TIC. Esters contribute to the fruity aroma of peaches [45]. Ethyl hexanoate (in the range from 1.38% to 5.76% of TIC) and ethyl tiglate (between 2.78% and 5.4% of TIC) were present at the highest relative concentrations among the estimated esters. Although the amount of esters was predominant in all eight varieties, the relative TIC was two times smaller than the reported literature average [46]. This is most likely due to the specificity of orchard location, light availability, temperature, and season specificity, as well as ecological location.

The presence of butanoates (B) and hexanoates (H) confirms the ripeness of the studied varieties from the averages of 9.48 (B) and 3.89 (H), 10.5 (B) and 2.61 (H), and 7.19 (B) and 5.33 (H) of the TIC in peaches, flat peaches, and nectarines, respectively.

Seven hydrocarbons were identified, of which tetradecane and tridecane were dominant. The total content of hydrocarbons was in the range from 7.23 to 10.20% of the TIC among the peach varieties. Lu et al. [47] reported the presence of several hydrocarbons in peaches. Tetradecane is considered a creamy descriptor, whereas dodecane is a woody descriptor [48]. The authors reported the presence of (E)-2-nonen-1-ol, 2-methylpropyl acetate, ethyl butanoate, butyl acetate, 3-methyl-1-butyl acetate, ethyl pentanoate, ethyl hexanoate, hexyl acetate, methyl octanoate, and hexyl hexanoate [47]. Other researchers differentiate ethyl acetate as the major ester compound in both peaches and nectarines [49].

Limonene, linalool, and p-cymene are listed as key flavor compounds that form the characteristic aroma profile [27,50]. The predominant compound in the nectarine varieties was β-mycrene, while linalool was the most abundant in the peach varieties, bringing out the floral aroma in them. Terpenes contribute to a floral flavor of fruits [51], and a sweetening taste [20], and are the principal components in plant essential oils. Among the eight identified terpenes, limonene (0.60–3.26% TIC) and linalool (1.1–2.75% TIC) were in the highest relative concentrations. Linalool was also the major terpenoid compound in other peaches and nectarines [13]. Linalool is reported to possess a floral and citrus-like aroma [52]. Mycrene is a precursor of linalool and is reported as an usual representative in peaches and nectarines, characterizing their aroma with a woody note. The currently established results also prove that the more the linalool, the less the myrcene. Other authors also advocate the thesis that myrcene is found in higher amounts in nectarines [35], which is further supported by the current values.

Terpene compounds (i.e., linalool) and alcohols (i.e., 1-hexanol) are reported to be less abundant than aldehydes in apricots and were detected to decrease with ripening [53]. The relative presence of terpenes in the eight studied varieties was lower compared to the aldehydes, in accordance with the results mentioned above.

**Figure 1:**
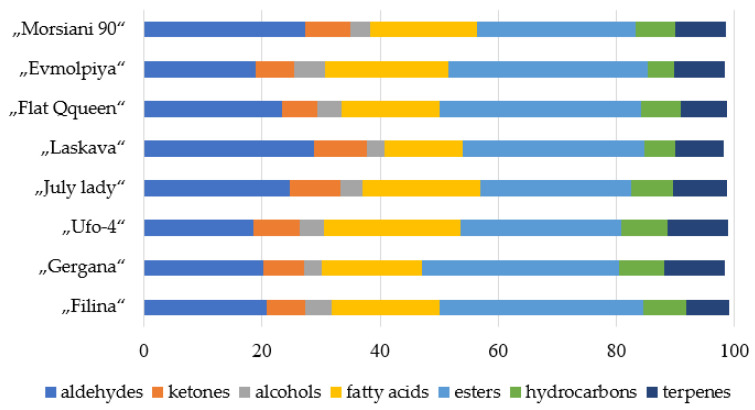
Distribution of volatile compounds according to their chemical families in the studied peach varieties.

The distribution of the main chemical families is presented in Figure 1. The volatile compounds were relatively uniformly distributed. The most abundant in all the varieties was esters, followed by aldehydes and fatty acids. The results are in line with those of Ortiz et al. [19], who stated that volatile esters often represent the major contribution in peaches (*Prunus persica* L.). However, fruit flavor is usually a complex mixture of a wide range of compounds [21]. The volatile composition provides significant information about the healthy composition of food, as it is synthesized from essential nutrients [54].

The “Laskava” variety had the highest relative total content of aldehydes (28.82% of the TIC) and ketones (8.87% of the TIC), while “Ufo-4” and “Flat Queen” had the lowest content. “Ufo-4” was the variety with the highest total relative quantity of fatty acids (23.05% of TIC) and hydrocarbons (7.83% of TIC). Alcohols and fatty acids were at the lowest total relative concentration in the “Laskava” variety, and the “Evmolpiya” variety had the highest total relative content of alcohols. “Gergana” and “Ufo-4” varieties were with the highest total relative content of terpenes (10.20% of TIC and 10.16% of TIC, respectively).

**Figure 2:**
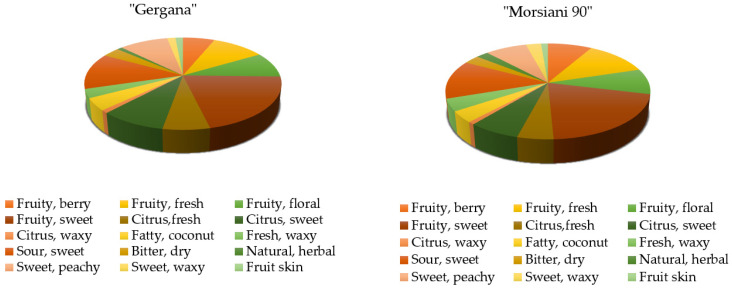
Flavor component distribution (%) in nectarine varieties.

Based on the odor descriptors, volatile compounds in peaches can be divided into several sensory groups, including green, fruity, and peach-like aromas [13]. To obtain a clear picture of the overall contribution of the identified compounds on the general flavor of the studied peaches, several figures were created. Figure 2 shows the odor/taste distribution [55] in the nectarine varieties based on the VOCs identified in the studied samples.

The two nectarine varieties had a dominant fruity, sweet vibe, as well as the sour sweet and sweet peachy descriptors. The “Gergana” variety can be seen as more citrus sweet and the “Morsiani 90” variety is more fruity fresh. Both varieties are considered sweet, but not as floral but fruity.

**Figure 3:**
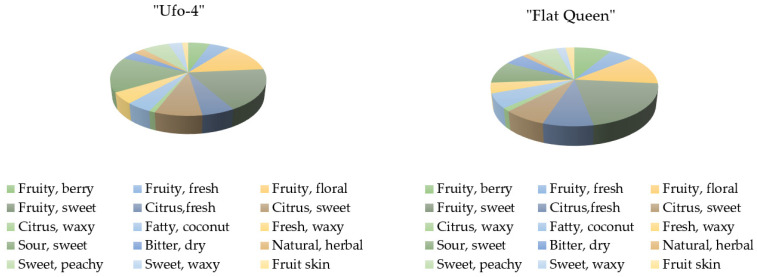
Flavor component distribution (%) in flat peach varieties.

The results for the two flat peach samples are given in Figure 3. The two flat peach samples are mostly fruity sweet, and floral. The “Ufo-4” variety possesses more sweet citrus scents than the “Flat Queen”, which is probably the reason for the more sour sweetness of “Ufo-4” compared to “Flat Queen”. This is quite distinctive for the white flesh peaches, as they are usually described as mildly acidic with a distinct sweet taste.

**Figure 4:**
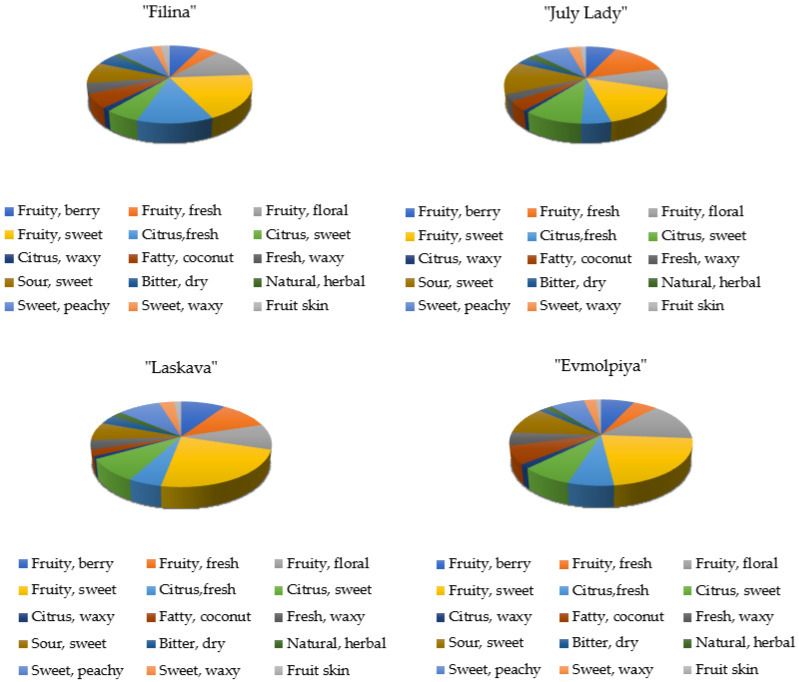
Flavor component distribution (%) in peach varieties.

When characterizing the four peach samples (Figure 4), it is evident that they are mainly fruity sweet.

The “Filina” variety has a moderate citrus fresh scent contributing to its overall flavor, while “July Lady” has a citrus sweet scent. “Evmolpiya” is more sour sweet than “Laskava”, while “Laskava” has a specific sweet waxy flavor.

### 2.2. Principal Component Analysis (PCA) and Hierarchical Cluster Analysis (HCA) of GC–MS Data

PCA is an exploratory technique that is typically used as a descriptive analysis for variable selection in a propensity model. The result quality fluctuates significantly by the number of factors or the factors-to-variables ratio. Considering the variables-to-observations ratio is not a good way to determine the required number of observations [56].

**Figure 5:**
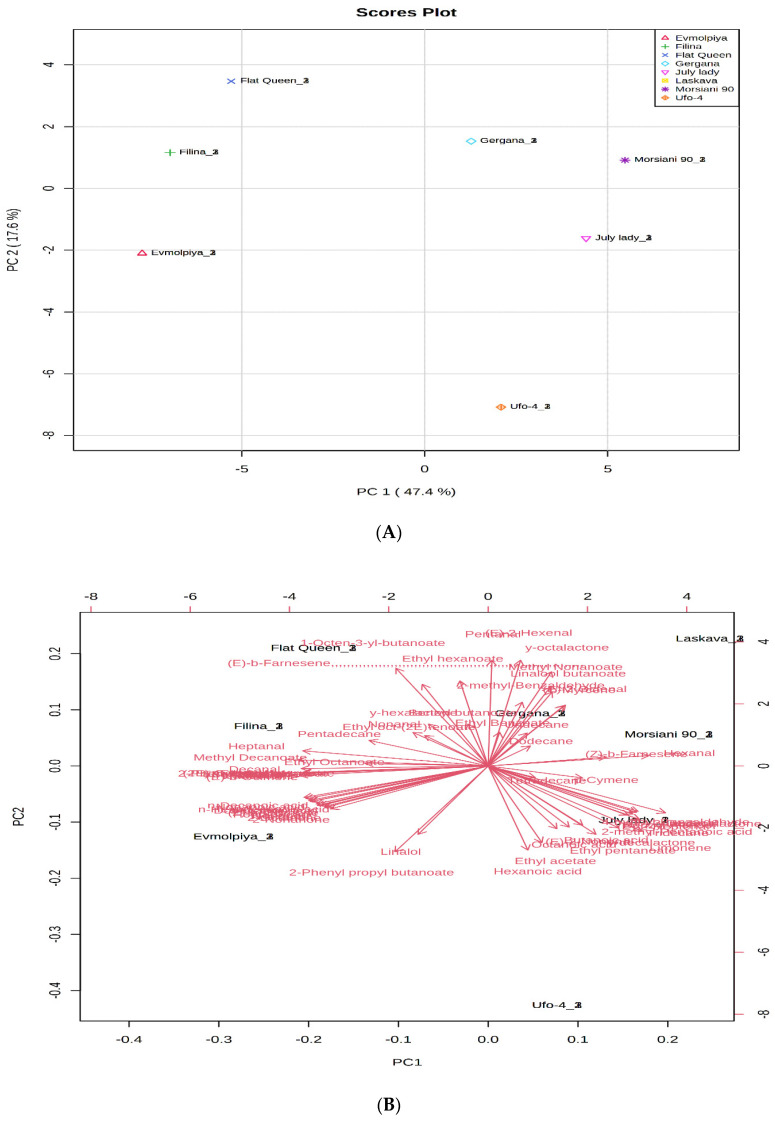
Principal component analysis (PCA) of GC–MS data for volatile compounds of peach (*Prunus persica* L.) varieties. (**A**) Principal component score plot for the eight peach varieties. (**B**) Eigenvector loading values of compounds identified in the eight peach varieties.

In order to confirm sample differences or similarities, principal component analysis (PCA) and hierarchical cluster analysis (HCA) of the volatile compounds identified were applied. According to the PCA plot obtained, the first two principal components PC1 (47.4%) and PC2 (17.6%) accounted for 65% of the total variance of all identified volatile compounds in the analyzed peach varieties (Figure 5).

(E)-2-Hexanal, pentanal, γ-octalactone, methyl nonanoate, dodecane, ethylbenzoate, hexanal, linalool, 2-methyl-benzaldehyde, β-myrcene, and (Z)-β-farnesene showed high positive loading scores in PC1 that distinguished the “Laskava”, “Morsiani 90”, and “Gergana” varieties from the other five. Volatile compounds with high negative scores in PC1 were 1-octen-3-yl-butanoate, ethyl hexanoate, (E)-β-farnesene, pentadecane, heptanal, methyl decanoate, nonanal, γ-hexalactone, and ethyl octanoate, which distinguished the “Flat Queen” and “Filina” varieties from the others. The “Evmolpiya” variety appeared clearly different from the other peach varieties, shown by the high negative loading values in PC2 of (E)-2-decenal, n-decanoic acids, 2-nonanone, linalool, and 2-phenyl propyl butanoate. Tetradecane, p-cymene, butanoic acid, 2-methyl-pentanoic acid, ethyl pentanoate, ethyl acetate, hexanoic acid, and limonene clearly differentiated the “July lady” and “Ufo-4” varieties from the other six.

No clear distinguishment between nectarines, peaches, and flat fruits could be stated based on the results. The early season fruits were quite similar to the late season and to the mid-season, and in reverse. Moreover, when clustering the metabolites in polar fractions (phenolic acids, amino acids, organic acids, sugar alcohols, carbohydrates, and saturated and unsaturated fatty acids [57]), different phytochemical similarities were reported.

**Figure 6:**
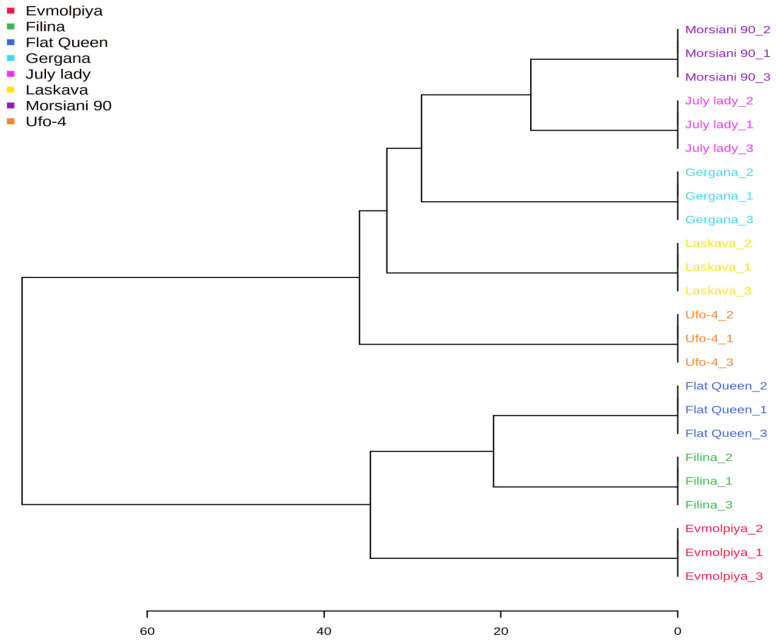
Clustering result of peach varieties shown as a dendrogram (by Euclidean distance measure, and Ward‘s clustering algorithm).

**Figure 7:**
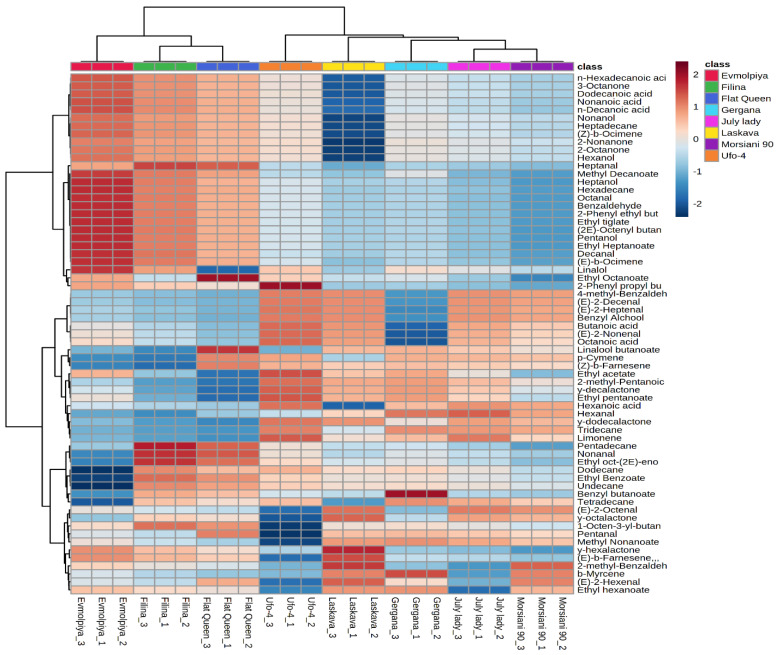
Clustering result of peach varieties shown as a heatmap. The color scale of the heat map ranges from dark brown (value, +2) to dark blue (value, −2). The values were normalized by log_10_ transformation.

HCA was performed to understand the relationships between the analyzed varieties. According to the dendrogram and heatmap obtained (Figure 6 and Figure 7), the “Filina” variety had the highest phytochemical similarity to “Flat Queen” and these were grouped in one cluster, with “Morsiani 90” and “July lady” grouped in another cluster. The observed clusters can be explained by the similar quantities of the identified metabolites. HCA also showed the highest diversity among the “Evmolpiya” and “Morsiani 90” varieties because of the significant differences in the quantities of the identified metabolites. According to the results obtained by PCA and HCA, the relative amounts of the identified volatile compounds differed between the studied varieties. In addition, many studies [13,15,51,58,59] also report that the volatile composition of peaches depends on the variety.

## 3. Materials and Methods

### 3.1. Fruit Material

Eight peach and nectarine varieties, from early to late harvesting dates, were the objects of analysis: “Filina” (peach, mid-June), “Ufo-4” (flat peach, white flesh, late June), “Gergana” (nectarine, late June), “Laskava” (peach, early August), “July Lady” (peach, mid-July), “Flat Queen” (flat peach, white flesh, early September), “Evmolpiya” (peach, mid-September), and “Morsiani 90” (nectarine, late September). All were grown on the same plantation in the 2020 season. “Filina”, “Gergana”, “Laskava”, and “Evmolpiya” are Bulgarian varieties created at the Fruit Growing Institute, Plovdiv, through interspecific hybridization. “Flat Queen”, “Morsiani 90”, “July Lady”, and “Ufo-4” are introduced varieties. No bactericides were applied to plants during testing. The undamaged peach, nectarine, and flat fruit were harvested at full ripeness, corresponding to their size, skin color, firmness and total soluble solids values. Fruit on the trees was considered ripe when the growth of the fruit had stopped and the fruit began to soften, exhibited a yellow or orange ground color (which is also specific to each variety), and was easily detached.

### 3.2. Headspace-Solid Phase Micro Extraction (HS-SPME) and Gas Chromatography–Mass Spectrometry Analysis (GC–MS)

For headspace sampling, a 2 cm SPME fiber assembly Divinylbenzene/Carboxen/Polydimethylsiloxane (DVB/CAR/PDMS, Supelco, Bellefonte, PA, USA) was used.

The HS-SPME technique was used for the extraction of the peach volatile according the procedure described by Uekane et al. [60]. The sampling procedure was automatically performed with a G1888 Network Headspace Sampler that was integrated on-line with the corresponding GC–MS system. An Agilent 7890A GC unit coupled to an Agilent 5975C MSD and a DB-5ms (30 m × 0.25 mm × 0.25 μm) column were used to analyze the volatile compounds in all investigated samples. The oven temperature program was as follows: from 40 °C (hold 1 min) to 250 °C (hold 5 min) at 2 °C/min; carrier gas: helium with flow rate: 1.0 mL/min; transfer line temperature: 270 °C; ion source temperature: 200 °C, EI energy: 70 eV, mass range: 50 to 550 m/z at 1.0 s/decade.

AMDIS software, version 2.64 (Automated Mass Spectral Deconvolution and Identification System, NIST, Gaithersburg, MD, USA) aided in the reading of the obtained mass spectra and the identification of the metabolites. For identification, the separated compounds were compared to the GC–MS spectra and Kovats retention index (RI) of reference compounds in the Golm Metabolome Database (http://csbdb.mpimp-golm.mpg.de/csbdb/gmd/gmd.html, accessed on 25 August 2021) and NIST’08 database (NIST Mass Spectral Database, PC-Version 5.0, 2008 from National Institute of Standards and Technology, Gaithersburg, MD, USA). The 2.64 AMDIS software recorded the RIs of the compounds with a standard n-hydrocarbon calibration mixture (C8–C36, Restek, Teknokroma, Spain).

### 3.3. Statistical Analysis

PCA and HCA of GC–MS data were conducted using MetaboAnalyst, a web-based platform (www.metaboanalyst.ca, accessed on 17 September 2021) [61]. Three analyses were performed for each of the eight peach varieties. The concentrations of the identified compounds were employed for PCA. All zero values were replaced with a small value (half of the minimum positive values in the original data) assumed to be the detection limit. First, PCA was applied in order to calculate the eigenvector loading values and to identify the major statistically different components among the observations (samples). The GC–MS data were mean-centered and the PCA model was obtained at a confidence level of 95%. The GC–MS data were also subjected to HCA, which produced a dendrogram by Ward’s method of hierarchical clustering and Euclidean distance measurement between the analyzed samples. The values were normalized by log10 transformation.

## 4. Conclusions

Aroma is particularly important in sensory evaluation. The human nose is sophisticated enough to be able to distinct the odor and flavor volatiles but the association between VOCs and aroma/flavor perception is not straightforward. Identifying odor-active compounds is an important stepping stone in the study of volatiles.

No data on VOCs profile for the local (“Filina”, “Laskava”, “Gergana”, “Evmolpiya”) or introduced (“Flat Queen”, “Morsiani 90”, “July Lady”, “Ufo-4”) peach varieties are available in the scientific literature, which makes this the first report on the subject. In total, sixty-five volatile compounds were identified; aldehydes, esters, and fatty acids were predominant. The overall contribution of the identified compounds on the general flavor of the studied peaches was explored. Some of the main groups represented were sweet, fruity, and floral flavors.

According to the obtained results, the PCA and HCA of volatile compounds can be successfully applied for the metabolic chemotaxonomy of peaches.

## Figures and Tables

**Table 1:** Identified volatile compounds in peach varieties analyzed by GC–MS. The results are given as % of Total Ion Current *.

Compound	Flavor Contribution **	RI_lit_	RI_calc_	“Filina”	“Gergana”	“Ufo-4”	“July Lady”	“Laskava”	“Flat Queen”	“Evmolpiya”	“Morsiani 90”
**Aldehydes**											
Pentanal	FB	738	741	0.70*	1.17	0.25	1.09	1.14	1.54	0.80	0.99
Hexanal	FFr	800	798	1.95	6.55	3.20	7.13	4.40	2.68	2.24	5.57
(E)-2-Hexenal	N/A	849	850	2.83	4.00	1.35	2.04	7.36	5.30	3.26	6.40
Heptanal	CF	907	909	4.35	1.47	1.58	1.38	1.14	3.95	3.00	1.25
Benzaldehyde	FSw	948	946	0.71	0.49	0.53	0.46	0.48	0.65	0.82	0.42
(E)-2-Heptenal	FW	960	960	0.51	0.35	1.64	1.42	1.49	0.46	0.58	1.30
Octanal	CF	999	1000	1.06	0.73	0.79	0.68	0.71	0.97	1.22	0.62
(E)-2-Octenal	FW	1051	1047	1.43	0.98	0.59	3.12	3.26	1.30	1.65	2.83
2-methyl-Benzaldehyde	FB	1070	1073	0.83	0.57	0.53	0.46	1.71	0.76	0.96	1.49
4-methyl-Benzaldehyde	FB	1084	1085	0.24	0.16	1.44	1.25	1.31	0.21	0.27	1.14
Nonanal	CW	1102	1104	3.89	2.21	2.37	2.06	1.99	3.54	1.48	1.88
(E)-2-Nonenal	CW	1160	1159	1.57	1.07	2.42	2.10	2.20	1.42	1.80	1.91
Decanal	CW	1204	1205	0.39	0.27	0.28	0.25	0.26	0.35	0.44	0.23
(E)-2-Decenal	FW	1250	1253	0.31	0.22	1.50	1.30	1.36	0.29	0.36	1.18
*Total aldehydes*				*20.77*	*20.24*	*18.47*	*24.74*	*28.81*	*23.42*	*18.88*	*27.21*
**Ketones**											
3-Octanone	N/A	975	977	0.71	0.49	0.52	0.45	0.25	0.64	0.82	0.41
2-Octanone	NH	991	992	0.56	0.38	0.41	0.36	0.14	0.51	0.64	0.32
γ-hexalactone	FSw	1045	1045	0.29	0.20	0.21	0.19	0.42	0.26	0.33	0.17
2-Nonanone	FW	1090	1088	0.64	0.44	0.47	0.41	0.14	0.58	0.73	0.37
γ-octalactone	SW	1250	1251	1.78	1.22	0.58	2.24	2.80	1.62	1.04	2.04
γ-decalactone	SP	1461	1464	1.11	1.52	1.63	1.42	1.48	1.01	1.28	1.29
γ-dodecalactone	N/A	1673	1675	1.47	2.52	4.00	3.48	3.64	1.34	1.69	3.16
*Total ketones*				*6.56*	*6.77*	*7.82*	*8.55*	*8.87*	*5.96*	*6.53*	*7.76*
**Alcohols**											
Pentanol	SW	770	772	1.63	1.12	1.21	1.05	1.10	1.49	1.88	0.95
Hexanol	FFl	851	848	0.49	0.34	0.36	0.32	0.13	0.45	0.57	0.29
Heptanol	NH	920	921	0.74	0.51	0.55	0.47	0.50	0.67	0.85	0.43
Benzyl Alcohol	FFl	1035	1035	0.26	0.18	1.03	0.90	0.94	0.23	0.30	0.81
Nonanol	N/A	1149	1150	1.36	0.93	1.00	0.87	0.39	1.24	1.57	0.79
*Total alcohols*				*4.48*	*3.08*	*4.15*	*3.61*	*3.06*	*4.08*	*5.17*	*3.27*
**Fatty Acids**											
Butanoic acid	SW	759	760	1.99	1.36	3.31	2.88	3.01	1.81	2.29	2.62
2-methyl-Pentanoic acid	FSw	926	924	1.77	2.72	2.93	2.55	2.66	1.61	2.03	2.31
Hexanoic acid	SS	964	966	2.59	4.80	6.84	5.95	1.22	2.36	2.98	5.41
Octanoic acid	SW	1165	1166	1.74	1.19	2.55	2.21	2.31	1.58	2.00	2.01
Nonanoic acid	NH	1270	1272	2.98	2.05	2.20	1.91	1.20	2.71	3.43	1.74
*n*-Decanoic acid	CW	1367	1368	2.53	1.74	1.87	1.63	1.07	2.30	2.91	1.48
Dodecanoic acid	FC	1558	1559	3.25	2.23	2.40	2.08	1.18	2.95	3.74	1.89
*n*-Hexadecanoic acid	FC	1960	1960	1.30	0.89	0.96	0.83	0.49	1.18	1.49	0.76
*Total fatty acids*				*18.15*	*16.98*	*23.06*	*20.04*	*13.14*	*16.5*	*20.87*	*18.22*
**Esters**											
Ethyl acetate	FSw	607	610	1.60	1.86	1.99	1.73	1.81	1.46	1.84	1.58
Ethyl pentanoate	FSw	903	905	1.29	1.64	1.76	1.53	1.60	1.17	1.48	1.39
Ethyl tiglate	FFl	940	938	4.76	3.27	3.51	3.06	3.19	4.33	5.48	2.78
Ethyl hexanoate	FSw	998	886	3.99	5.76	1.59	1.38	5.63	3.63	4.59	4.90
Ethyl Heptanoate	FSw	1096	1097	2.08	1.43	1.54	1.34	1.40	1.89	2.40	1.21
Ethyl Benzoate	BD	1170	1173	3.58	2.45	2.64	2.30	2.40	3.25	1.11	2.09
Ethyl Octanoate	FSw	1195	1198	2.04	2.09	2.24	1.95	2.04	2.76	2.35	1.77
Methyl Nonanoate	FC	1226	1225	1.70	2.67	0.88	2.50	2.61	1.54	1.95	2.27
Ethyl oct-(2E)-enoate	FSk	1242	1240	1.55	1.07	1.15	1.00	1.04	1.41	0.79	0.91
1-Octen-3-yl-butanoate	FB	1280	1280	2.19	1.50	0.61	1.40	1.47	1.99	1.52	1.28
Methyl Decanoate	FFl	1320	1322	1.16	0.80	0.72	0.62	0.65	1.06	1.34	0.57
Benzyl butanoate	SP	1344	1345	1.24	2.36	0.76	0.66	0.69	1.13	0.43	0.60
(2E)-Octenyl butanoate	FB	1388	1385	1.63	1.12	1.20	1.05	1.09	1.48	1.87	0.95
Linalool butanoate	FFl	1423	1425	1.19	2.19	1.35	2.04	2.14	2.90	1.36	1.86
2-Phenyl ethyl butanoate	FFl	1435	1436	2.75	1.88	2.03	1.76	1.84	2.50	3.16	1.60
2-Phenyl propyl butanoate	FS	1482	1480	1.86	1.28	3.37	1.19	1.25	1.69	2.14	1.08
*Total esters*				*34.61*	*33.37*	*27.34*	*25.51*	*30.85*	*34.19*	*33.81*	*26.84*
**Hydrocarbons**											
Undecane	N/A	1098	1095	1.06	0.73	0.79	0.68	0.71	0.97	0.22	0.62
Dodecane	N/A	1200	1202	1.51	1.30	1.40	1.22	1.27	1.37	0.73	1.11
Tridecane	N/A	1302	1304	0.33	1.74	1.87	1.62	0.70	0.30	0.38	1.48
Tetradecane	FW	1400	1401	2.03	2.31	2.05	2.16	1.26	1.85	1.03	1.97
Pentadecane	FW	1497	1495	0.97	0.66	0.71	0.62	0.65	0.88	0.61	0.56
Hexadecane	N/A	1600	1601	0.52	0.36	0.39	0.34	0.35	0.48	0.60	0.31
Heptadecane	N/A	1701	1700	0.85	0.58	0.63	0.54	0.26	0.77	0.97	0.49
*Total hydrocarbons*				*7.27*	*7.68*	*7.84*	*7.18*	*5.2*	*6.62*	*4.54*	*6.54*
**Terpenes**											
β-Myrcene	FW	980	985	1.13	2.53	0.96	0.84	2.15	1.02	1.29	2.15
p-Cymene	CF	1018	1020	0.15	1.06	1.14	0.99	0.36	1.40	0.18	0.90
Limonene	CS	1024	1022	0.66	1.96	3.26	2.83	1.49	0.60	0.76	1.67
(Z)-β-Ocimene	NH	1035	1036	0.79	0.54	0.58	0.51	0.25	0.72	0.91	0.46
(E)-β-Ocimene	NH	1042	1041	1.42	0.97	1.04	0.91	0.90	1.29	1.63	0.83
Linalol	FFl	1093	1094	2.22	1.86	2.00	1.74	1.48	1.10	2.75	1.58
(Z)-β-Farnesene	CS	1440	1443	0.26	0.87	0.93	0.81	0.80	1.15	0.30	0.74
(E)-β-Farnesene	N/A	1452	1455	0.60	0.41	0.24	0.39	0.84	0.55	0.69	0.35
*Total terpenes*				*7.23*	*10.2*	*10.15*	*9.02*	*8.27*	*7.83*	*8.51*	*8.68*

* RI—Kovats retention index; ** FB—fruity, berry; FSw—fruity, sweet; FFr—fruity, fresh; FFl—fruity, floral; CW—citrus, waxy; CF—citrus, fresh; CS—citrus, sweet; SS—sour, sweet; SP—sweet, peachy; SW—sweet, waxy; FC—fatty, coconut; FW—fresh, waxy; BD—bitter, dry; NH—natural, herbal; FSk—fruit skin; N/A—not available.

## Data Availability

The data presented in this study are available on request from the corresponding author.
